# Multidisciplinary recommendations for diagnosis and treatment of foot problems in people with rheumatoid arthritis

**DOI:** 10.1186/s13047-018-0276-z

**Published:** 2018-07-04

**Authors:** Marloes Tenten-Diepenmaat, Marike van der Leeden, Thea P. M. Vliet Vlieland, Joost Dekker, on behalf of the RA Foot Expert Group, Dirkjan van Schaardenburg, Wiepke Drossaers-Bakker, Bianca Lourens, Rianne van Berkel, Patricia Smith-van der Meijde, Els van Buuren, Leo Roorda, Antal Sanders, Huub van der Heide, Kirsten Veenstra, Sabine van Vliet-Koppert, Elleke Huijbrechts, Michel Boerrigter, Rob Verwaard, Arthur Arets, Willem Seves, Toos Mennen, Maya Ribbink, Bertha Maat, Wijnanda Hoogland

**Affiliations:** 1 Amsterdam Rehabilitation Research Center | Reade, Amsterdam, the Netherlands; 2 Department of Rehabilitation Medicine, VU University Medical Center, Amsterdam, the Netherlands; 3 Amsterdam Public Health research institute, VU University Medical Center, Amsterdam, the Netherlands; 4 Department of Orthopaedics, Rehabilitation and Physical Therapy, Leiden University Medical Center, Leiden, the Netherlands

**Keywords:** Rheumatoid arthritis, Foot, Recommendations, Multidisciplinary

## Abstract

**Background:**

Foot problems in people with rheumatoid arthritis (RA) are highly prevalent and have a substantial impact on quality of life. Healthcare professionals from various professions can be involved in the management of these foot problems. There is currently no consensus on optimal management. Therefore, the aim of the present study was to develop multidisciplinary recommendations for the management of foot problems in people with RA in the Netherlands.

**Methods:**

The recommendations were based on research evidence and consensus among experts, following published strategies for the development of practice recommendations. The expert group was composed of 2 patients and 22 experienced professionals (rheumatologists, rehabilitation physicians, orthopaedic surgeons, specialized nurses, podiatrists, orthopaedic shoe technicians, pedicurists, and researchers) in the Netherlands. For each developed recommendation i) the level of evidence was determined, and ii) the level of agreement (among the expert group) was set by an anonymous voting procedure using a numeric rating scale. The mean and range of the level of agreement for each recommendation was calculated. A recommendation was approved when ≥70% of the expert group voted an NRS-agreement ≥7.

**Results:**

In total, 41 recommendations were developed. Two recommendations concerned a framework for diagnosis and treatment. Thirty-nine recommendations on foot care were developed: seven on diagnosis (including check-ups of feet and shoes and diagnostic imaging), 27 on treatment (including corticosteroid injections, foot surgery, therapeutic shoes, foot orthoses, exercise therapy, toe-orthoses and toenail-braces, treatment of toenails and skin), four on communication, and one on organisation of RA-related footcare. All recommendations were approved by the expert group. The percentage score of NRS-agreement ≥7 ranged from 80 to 100%.

**Conclusions:**

These are the first published multidisciplinary recommendations specific to the management of foot problems in people with RA. Multidisciplinary recommendations can provide guidance in timely referrals and access to adequate footcare. More research is needed to strengthen the evidence on diagnosis and treatment of RA-related foot problems. These national recommendations may be a first step towards developing international multidisciplinary recommendations for the management of foot problems in RA.

**Electronic supplementary material:**

The online version of this article (10.1186/s13047-018-0276-z) contains supplementary material, which is available to authorized users.

## Background

Approximately 90% of patients with rheumatoid arthritis (RA) experience foot problems, such as pain, swelling, and stiffness, during the course of the disease [1–4]. In a more advanced stage of RA, joint damage and foot deformities may occur [5]. In addition, dermatological abnormalities and reduced sensitivity are more frequent in people with RA compared with the healthy population [6]. Foot involvement in RA may result in an abnormal foot function, limitations in daily activities such as standing and walking, and a reduced quality of life [7, 8].

It seems important to start management of foot problems in an early disease stage to reduce pain and activity limitations, and to prevent deterioration of foot function [9]. The primary treatment of foot problems related to disease activity is systemic medication. In addition, local pharmacological treatment (corticosteroid injections), surgical treatment, or conservative treatment (such as foot orthoses, therapeutic shoes, removal of callosities) can be applied [10]. Apart from rheumatologists and orthopaedic surgeons, healthcare professionals from various professions can be involved. In the Netherlands there is a role for rehabilitation physicians, specialized nurses, podiatrists, orthopaedic shoe-technicians, and pedicurists in the management of RA-related foot problems [10]. A multidisciplinary approach is necessary in order to offer treatment with adequate content and timing for the individual patient [9, 11, 12].

Despite the high prevalence of foot problems in RA, underuse of foot care seems apparent. In a specialized center for rheumatology and rehabilitation in the Netherlands only 40% of the people with RA received specific footcare (10), while in primary care foot problems appear to be treated even less. Among healthcare professionals there is often limited expertise in detecting and managing RA-related foot problems, as shown in a survey among podiatrists in New South Wales [13]. Similarly, among patients there is limited knowledge of the possibilities of, and access to, footcare [13, 14]. A survey among patients in the Netherlands showed that 94% of the patients reported insufficient knowledge about the content and accessibility of health care services [14].

Multidisciplinary recommendations provide guidance on timely referrals and access to adequate footcare. Previously published guidelines were recently critically appraised by Hennessy et al. [15]. In their work, 24 guidelines recommending management of RA-related foot problems were identified. Of these guidelines, only five (general) guidelines were of high quality and recommended for use without modifications. Moreover, only a small section of the guidelines (ranging from one sentence to one page) were foot-specific [15]. Only two published guidelines were foot and ankle specific, one of low [12] and one of high [11] quality [15]. Additionally, these guidelines are monodisciplinary (podiatry) [11, 12]. The aim of the present study was to develop multidisciplinary recommendations and frameworks for the diagnosis and treatment of foot problems in people with RA.

## Methods

Recommendations for management of RA-related foot problems were based on research evidence and consensus among experts (healthcare providers, patients, and researchers). The methodology for the development of the recommendations was based on published strategies for the development of practice recommendations [16, 17]. The expert group was composed of patients (experienced with foot problems and related treatments) and experienced professionals (from leading expertise centres or nominated by professional bodies) of several professions involved in RA footcare in the Netherlands. The expert group included two patients, two rheumatologists, two rehabilitation physicians, three orthopaedic surgeons, four specialized nurses, two podiatrists, three orthopaedic shoe technicians, two pedicurists, and four researchers (the core members; MTD, MvdL, TPMVV and JD). Three expert group meetings took place between February 2015 and July 2016.

There were four phases in the development of the recommendations. A detailed description of the steps taken in the different phases, is given in Table 1. In the first phase, definitive research questions and semi-definitive frameworks for diagnosis and treatment were developed based on: i) a preliminary literature search, ii) semi-structured interviews with four persons with RA, iii) a field consultation among 39 RA footcare professionals (medical doctors/allied healthcare professionals), iv) discussion within the core members, and v) discussion with the experts during the first expert group meeting.

**Table 1:** Development of the recommendations

Phase 1. *Development of research-questions and semi-definitive frameworks for diagnosis and treatment*	
a	Preliminary literature search in books, protocols and review articles
b	Semi-structured interviews with 4 RA patients experienced with foot problems and related treatments
c	Field consultation among 39 RA footcare professionals (medical doctors/allied healthcare professionals) by assessing a semi-structured interview (*n* = 6) or by using a questionnaire during an expert meeting (*n* = 33). The overall question to be answered: *“Which questions would you like to see answered by the recommendations? Regarding to your field of expertise (diagnostics and treatment) and in the context of a multidisciplinary approach”*
d	Draft research questions and draft frameworks (for diagnosis and treatment) were developed, by the core members of the expert group (MTD, MvdL, TPMVV and JD), based on the results of point a-c.
e	Discussion with the experts on the draft research questions and frameworks, during the first expert group meeting.
f	Refining draft research questions and frameworks into definitive research questions and semi-definitive frameworks with the expert group, during the first expert group meeting.
Phase 2. *Development of draft recommendations*	
g	A search strategy was developed for each research question (see Additional file 1). Literature was searched in PubMed by MTD. The available (systematic) reviews on the subject of interest were used. When no (systematic) review were available, core publications (according to the expert group) were used.
h	Draft recommendations were formulated (by the core members) based on the literature found at point g.
Phase 3. *Development of definitive recommendations and frameworks with a level of evidence*	
i	Discussion with the experts on the draft recommendations and semi-definitive frameworks, during the second expert group meeting and 2 email-rounds.
j	Refining draft recommendations and semi-definitive frameworks into definitive recommendations and frameworks, during the second expert group meeting and 2 email-rounds.
k	Determining the level of evidence for each definitive recommendation/framework according to “Evidence-Based Guideline Development” of the Quality Institute for Public Healthcare in The Netherlands. Five levels of evidence were distinguished (ranging from 1 to 4b). When a recommendation was based on a review or guideline, the level of evidence reported in the review/guideline was used. If the level of evidence was not reported, the original sources were retrieved (individual studies/ expert opinion).
Phase 4. *Determining the level of agreement for the definitive recommendations and frameworks*	
l	During the third expert group meeting an anonymous voting procedure was followed. For each recommendation/framework a numeric rating scale for agreement (NRS-agreement) from 0 (total disagreement) to 10 (total agreement) was assessed.
m	The mean and range of the level of agreement for each recommendation was calculated. A recommendation was approved when ≥70% of the expert group voted an NRS-agreement ≥7.

In the second phase, draft recommendations were formulated (by the core members) based on relevant literature, to answer the research questions. Literature was searched in PubMed by MTD. Additional file 1 gives an overview of the search-details. The available (systematic) reviews on the subject of interest were used to develop the draft recommendations. When no (systematic) review was available, core publications (according to the expert group) or available guidelines were used.

In the third phase definitive recommendations and frameworks with a level of evidence were developed. The draft recommendations and semi-definitive frameworks were discussed with the experts during a second expert meeting and by email rounds. The draft recommendations and semi-definitive frameworks were refined into definitive recommendations and frameworks. For each final recommendation/framework, the level of evidence was determined. The methodological quality was determined according to the “Evidence-Based Guideline Development” of the Quality Institute for Public Healthcare in The Netherlands, as shown in Table 2 [18]. Five levels of evidence were distinguished (ranging from 1 to 4b), as shown in Table 3. When a recommendation was based on a review or guideline, the level of evidence reported in the review/guideline was used. If the level of evidence was not reported, the original sources were retrieved (individual studies/ expert opinion).

**Table 2:** EBRO classification of methodological quality of individual studies [18]

A1	Systematic review of at least two independent studies of A2-level
A2	Randomized double-blind controlled clinical trial of good quality and of sufficient size
B	Controlled trial but not with all the characteristics as mentioned under A2
C	Non-controlled studies
D	Expert opinion

**Table 3:** Level of evidence

	Evidence is based on
1	Research of level A1 or at least 2 independently conducted studies of level A2
2	1 study of level A2 or at least 2 independently conducted studies of level B
3	1 study of level B or C
4a	Expert opinion described in the literature
4b	Opinion of the expert group

In the fourth phase, the level of agreement for each recommendation/framework was set by an anonymous voting procedure during the third expert meeting. A numeric rating scale for agreement (NRS-agreement) from 0 (total disagreement) to 10 (total agreement) was used. The mean and range of the level of agreement for each recommendation was calculated. A recommendation was approved when ≥70% of the expert group voted a NRS-agreement ≥7 [19].

## Results

Fifteen research questions were developed during phase 1. Two (out of 15) research questions concerned the quality of the developed frameworks for diagnosis and treatment. These frameworks and answers to the related research questions were based on expert opinion. The answers of 13 (out of 15) research questions were based on both literature and expert opinion. Additional file 1 shows an overview of the developed research questions and the answering methods. The developed frameworks were reflected in two recommendations. Furthermore, 39 care-related recommendations were developed: seven on diagnosis, 27 on treatment, four on communication and one on organisation of footcare. All recommendations were approved. Tables 4, 5, 6, 7, 8, 9 give an overview of the developed recommendations with references to the literature used, the level of evidence, and the level of agreement. The percentage score of NRS-agreement ≥7 ranged from 80 to 100%.

**Table 4:** Recommendations on the framework for diagnosis and the framework for treatment of RA-related foot problems

	LoE	Ref	LoA
The “Framework for diagnosis of RA-related foot problems” (Fig. 1) provides an overview of the different objectives in detection, diagnosis, and monitoring of foot problems in people with RA, as well as the corresponding instruments.	4b	n/a	9.2 (7–10)
The “Framework for treatment of RA-related foot problems” (Fig. 2) provides an overview of the potential treatment per diagnostic outcome.	4b	n/a	9.1 (6–10)

*LoE* Level of Evidence for the recommendations: (1) research of level A1 or at least 2 independently conducted studies of level A2, (2) 1 study of level A2 or at least 2 independently conducted studies of level B, (3) 1 study of level B or C, (4a) expert opinion described in the literature, (4b) opinion of the expert group*. Ref.* references*, LoA* Level of Agreement for the recommendations, Numeric Rating Scale from 0 (total disagreement) to 10 (total agreement) reported as mean (range)*. n/a* not applicable

**Table 5:** Recommendations on check-ups of feet and shoes

	LoE	Ref	LoA
Rheumatologists and nurses specialised in rheumatology should perform regular feet check-ups. These check-ups should include, at least, patient history of foot disease, foot inspection, and palpation of foot joints for the detection of swelling and pain.	4b	n/a	9.2 (8–10)
Over-the-counter shoes should have, at least, sufficient room in the toe box and a stiff sole allowing a heel-to-toe gait. *The following additional shoe features may be important, depending on the foot conditions and wishes of the patient: i) light weight; ii) spacious, adjustable, and easy to close in-step/heel girth; iii) strong, raised, and padded heel part; iv) inflection point at the MTP joints; v) adequate length and width, measured in standing position; vi) no seams on the inside; vii) removable insoles so that custom-made foot orthoses can be placed in it.**	*3**4a	* [46] ** [32, 33]	9.3 (7–10)

*LoE* Level of Evidence for the recommendations: (1) research of level A1 or at least 2 independently conducted studies of level A2, (2) 1 study of level A2 or at least 2 independently conducted studies of level B, (3) 1 study of level B or C, (4a) expert opinion described in the literature, (4b) opinion of the expert group*. Ref.* references. *LoA* Level of Agreement for the recommendations: Numeric Rating Scale from 0 (total disagreement) to 10 (total agreement) reported as mean (range)*. n/a* not applicable. * refers to the first part of the recommendation with corresponding level of agreement and references. ** refers to the second part of the recommendation with corresponding level of agreement and references

**Table 6:** Recommendations on diagnostic imaging

	LoE	Ref	LoA
For the detection of joint damage in the feet, a non-weight-bearing X-ray in anterior-posterior (AP) direction is the preferred method.	4b	n/a	8.6 (0–10)
For the detection of joint deformity and malalignment of the foot, a weight-bearing X-ray in anterior-posterior (AP) and lateral directions is the preferred method.	4b	n/a	9.6 (7–10)
Ultrasonography can be applied in the diagnosis of inflammation of joints* and soft tissue**.	2	* [47, 48] ** [49, 50]	9.4 (7–10)
When clinical examination is inconclusive in the diagnosis of inflammation of joints and soft tissue, ultrasonography should be considered. *When ultrasonography is inconclusive, additional diagnostic imaging (MRI or CT scan) can be considered.**	*4a**4b	* [51] ** n/a	9.2 (8–10)

*LoE* Level of Evidence for the recommendations: (1) research of level A1 or at least 2 independently conducted studies of level A2, (2) 1 study of level A2 or at least 2 independently conducted studies of level B, (3) 1 study of level B or C, (4a) expert opinion described in the literature, (4b) opinion of the expert group*. Ref.* references*. LoA* Level of Agreement for the recommendations: Numeric Rating Scale from 0 (total disagreement) to 10 (total agreement) reported as mean (range)*. n/a* not applicable. * refers to the first part of the recommendation with corresponding level of agreement and references. ** refers to the second part of the recommendation with corresponding level of agreement and references

**Table 7:** Recommendations on medical treatment

	LoE	Ref	LoA
Corticosteroid injections can be applied in joints and soft tissue of the foot in the treatment of local arthritis and synovitis.*Corticosteroid injections may also be applied in the treatment of tendinitis and pain.**	*2 **4a/b	* [52] [53] ** [32, 54, 55]	8.7 (7–10)
A corticosteroid injection conducted by ultrasonography (if available) is preferred, because this may result in a more accurate determination of the location of the injection.	4b	n/a	9.4 (7–10)
Early in the treatment process, consultation by an orthopaedic surgeon should be considered. Surgical intervention should be considered when the following foot conditions do not respond to conservative therapy: i) persistent pain and stiffness, ii) > 6 months of synovitis in foot and ankle joints, iii) tenosynovitis or tendon ruptures, iv) malalignment of the foot (e.g., hammer toes) causing mobility limitations and pain or problems finding adequate shoes, v) returning callosity/clavus, vi) wounds/(pre)ulcers, and vii) osteomyelitis/septic arthritis.	4a/b	[27, 32, 56]	9.1 (6–10)
Resection arthroplasty of the MTP joints can be applied to improve joint mobility and to reduce pain, forefoot plantar pressure, and problems finding well-fitting shoes.* In severe malalignments of the toes or damage to the MTP joints, resection arthroplasty is preferred. Without severe malalignments/damage, a MTP joint-preserving surgical technique can be considered.**	*3 **4a	* [57] ** [56]	8.9 (6–10)
An arthrodesis of the MTP1 joint can be performed to reduce pain and improve the weight-bearing capacity of the forefoot.	3	[37]	9.1 (7–10)
When surgical treatment of the hindfoot is necessary, arthrodesis of the subtalar joint is preferred. For flat feet, an additional arthrodesis of the calcaneocuboid joint and talonavicular joint should be considered (triple arthrodesis).	4a	[39]	8.9 (6–10)
In the treatment of severe pain and damage of the tibiotalar joint, an arthrodesis of the tibiotalar joint or an ankle prosthesis can be applied.* An arthrodesis is preferred, provided that the Chopart-joint-line is intact and the status of other joints does not form a contraindication. An ankle prosthesis can be considered when preservation of mobility in the tibiotalar joint is important (according to the patient) and the preoperative status of the patient does not form a contra-indication.**	*1 **4b	* [58] ** n/a	9.0 (7–10)

*LoE* Level of Evidence for the recommendations: (1) research of level A1 or at least 2 independently conducted studies of level A2, (2) 1 study of level A2 or at least 2 independently conducted studies of level B, (3) 1 study of level B or C, (4a) expert opinion described in the literature, (4b) opinion of the expert group*. Ref.* references*, LoA* Level of Agreement for the recommendations: Numeric Rating Scale from 0 (total disagreement) to 10 (total agreement) reported as mean (range). *n/a* not applicable. * refers to the first part of the recommendation with corresponding level of agreement and references. ** refers to the second part of the recommendation with corresponding level of agreement and references

**Table 8:** Recommendations on conservative treatment

	LoE	Ref	LoA
Technical adaptations to over-the-counter shoes can reduce pain and improve physical functioning.* These adaptations can be prescribed in patients with abnormal foot function, foot joint damage/deformity, or malalignment of the feet, provided that the feet fit in over-the-counter shoes.**	*3**4b	* [59] **n/a	9.3 (8–10)
Ready-made therapeutic shoes with extra depth, support, incorporated inlays, and optional technical adaptation can reduce forefoot plantar pressure and foot pain and improve gait characteristics, physical functioning, and health-related quality of life.* These ready-made shoes can be prescribed in patients with i) abnormal foot function, foot joint damage/deformity, or malalignment of the feet, and ii) feet that do not fit in over-the-counter shoes, but for whom custom-made shoes are not indicated.**	*3 **4b	* [46, 60–64] **n/a	9.3 (7–10)
Custom-made therapeutic shoes can reduce pain and improve physical functioning.* These custom-made shoes can be prescribed in patients with i) abnormal foot function, foot joint damage/deformity, or malalignment of the feet, and ii) feet that do not fit in over-the-counter shoes or ready-made therapeutic shoes.**	*3 **4b	* [25] **n/a	9.5 (8–10)
Custom-made therapeutic shoes should be worn all day, after a habituation period.	3	[25]	8.5 (0–10)
Foot orthoses are recommended in patients with abnormal foot function, when adequate over-the-counter shoes are insufficient in reducing foot symptoms.	4a/b	[27–31]	9.0 (2–10)
Foot orthoses in adequate shoes can reduce forefoot plantar pressure and pain.	1	[28, 30]	9.4 (7–10)
The function of foot orthoses should be assessed in relation to the patient’s footwear, due to the interaction between the two.	3	[60]	9.3 (8–10)
Rigid foot orthoses are recommended in feet with correctable malalignment, to control the position of the feet during weight-bearing.	4a	[27, 29, 32, 33]	8.9 (7–10)
Total contact foot orthoses are recommended in feet with uncorrectable malalignment or fragile skin. The material used for the production of total contact foot orthoses depends on the required characteristics of the foot orthoses.	4a/b	[29, 32]	9.0 (6–10)
General exercise therapy is recommended according to the Dutch KNGF Guideline for Physical Therapy in Patients with Rheumatoid Arthritis.	1	[34]	9.1 (7–10)
Exercise therapy specific to the foot and ankle can include i) strengthening exercises for the intrinsic foot muscles and *M. tibialis* posterior; ii) active stretch exercises for the plantar fascia, achilles-tendon, and peroneal muscles; and iii) active exercises to improve joint mobility.	4a	[33]	8.8 (7–10)
A silicone toe orthosis can be used in the treatment of malalignment of toes and secondary pain or high pressure.	3	[65]	9.2 (7–10)
In the prescription of a silicone toe orthosis, the following factors should be considered: i) a sensibility disorder or peripheral artery disease; ii) a skin defect on the foot of interest; and iii) sufficient room in the shoe for wearing the toe orthosis.	4a/b	[36]	9.3 (8–10)
A toenail brace can be used in the treatment of an ingrowing or ingrown toenail. ^	2	[66, 67]	8.8 (5–10)
In the prescription of a toenail brace, the following factors should be considered: i) a sensibility disorder or peripheral artery disease; ii) a skin defect, inflammation, or onycholysis on the toe of interest; and iii) the use of biologicals.	4a/b	[36]	9.3 (7–10)
When a fungal nail or mycosis of the skin is detected, treatment should be started to prevent ulcers and secondary bacterial infections.	4a/b	[32]	9.0 (7–10)
Pressure and shearing forces should be normalised in feet with hyperkeratotic lesions. For normalisation of pressure and shearing forces, i) an individual shoe- and sock advice can be given; or ii) foot orthoses, silicone toe orthosis, technical adaptations to over-the-counter shoes, ready- or custom-made therapeutic shoes, or a provisional therapy (e.g., felt padding or taping) can be prescribed.	4a/b	[32, 36]	9.0 (6–10)
Excessive hyperkeratotic lesions should be treated. During the treatment the following factors should be considered: i) a sensibility disorder or peripheral artery disease, and ii) fragile skin, plantar bursa, and prominent metatarsal heads on the foot of interest.	4a/b	[32, 36]	9.1 (7–10)
When an (pre-)ulcer or infection is detected, the treating physician should be consulted.	4a/b	[32]	9.2 (6–10)
In wound-care, a provisional therapy (e.g., felt padding) can be applied to reduce pressure. When material with an adhesive layer is used, fragile skin should be taken into consideration.	4a	[32]	8.8 (7–10)

*LoE* Level of Evidence for the recommendations: (1) research of level A1 or at least 2 independently conducted studies of level A2, (2) 1 study of level A2 or at least 2 independently conducted studies of level B, (3) 1 study of level B or C, (4a) expert opinion described in the literature, (4b) opinion of the expert group*. Ref.* references*. LoA* Level of Agreement for the recommendations: Numeric Rating Scale from 0 (total disagreement) to 10 (total agreement) reported as mean (range)*. n/a* not applicable. ^ based on literature not specific for RA. * refers to the first part of the recommendation with corresponding level of agreement and references. ** refers to the second part of the recommendation with corresponding level of agreement and references

**Table 9:** Recommendations on communication and organisation of RA-related footcare

	LoE	Ref	LoA
Regular consultation and shared decision-making between the patient and healthcare professional should be included in RA-related footcare and should be customised to the individual patient.	4b	n/a	8.8 (5–10)
Individual shoe-advice to people with RA with foot problems is essential and should include information on fit, cosmetics, function, durability and correct use of the shoes.	4a/b	[32, 33, 35]	9.4 (8–10)
Footcare in patients with RA should include patient education.* Patient education may consist of preventive and curative care.**	*1 **4b	* [68] **n/a	9.6 (7–10)
Patient education on preventive care for RA-related foot problems should contain information about i) the cause and course of RA and RA-related foot disease; ii) recognition of infection and increased disease activity (systemic and local); iii) footcare and hygiene; iv) recognition and use of adequate footwear (for indoors and outdoors); v) timely consultation by a healthcare professional in the case of foot infection, symptoms of increased disease activity, pain, problems finding adequate footwear, and skin and nail conditions; and vi) the healthcare professional who may be consulted for a specific indication.	4a	[11, 32, 33, 35]	9.3 (8–10)
Patient education on curative care for RA-related foot problems should contain information about i) the treatment strategy (short and long term); ii) the importance of treatment adherence and compliance; iii) the expected treatment results according to pain, physical functioning, activities, and participation; iv) the possible adverse events; and v) costs and reimbursement of the treatment.	4a	[33, 35, 39, 51, 69]	9.2 (7–10)
A multidisciplinary approach in management of RA-related foot problems is recommended. The diagnosis and treatment of RA-related foot disease consists of different aspects, which require the expertise of several disciplines.	4a/b	[11, 32]	9.6 (8–10)

*LoE* Level of Evidence for the recommendations: (1) research of level A1 or at least 2 independently conducted studies of level A2, (2) 1 study of level A2 or at least 2 independently conducted studies of level B, (3) 1 study of level B or C, (4a) expert opinion described in the literature, (4b) opinion of the expert group. *Ref.*references*. LoA* Level of Agreement for the recommendations: Numeric Rating Scale from 0 (total disagreement) to 10 (total agreement) reported as mean (range)*. n/a* not applicable. * refers to the first part of the recommendation with corresponding level of agreement and references. ** refers to the second part of the recommendation with corresponding level of agreement and references

### Frameworks for diagnosis and treatment

A framework for diagnosis and a framework for treatment were developed by using the terminology of the International Classification of Functioning, Disability and Health (ICF) of the World Health Organization [20]. (Table 4) provides an overview of the developed recommendations on the frameworks for diagnosis and treatment.

**Fig. 1:**
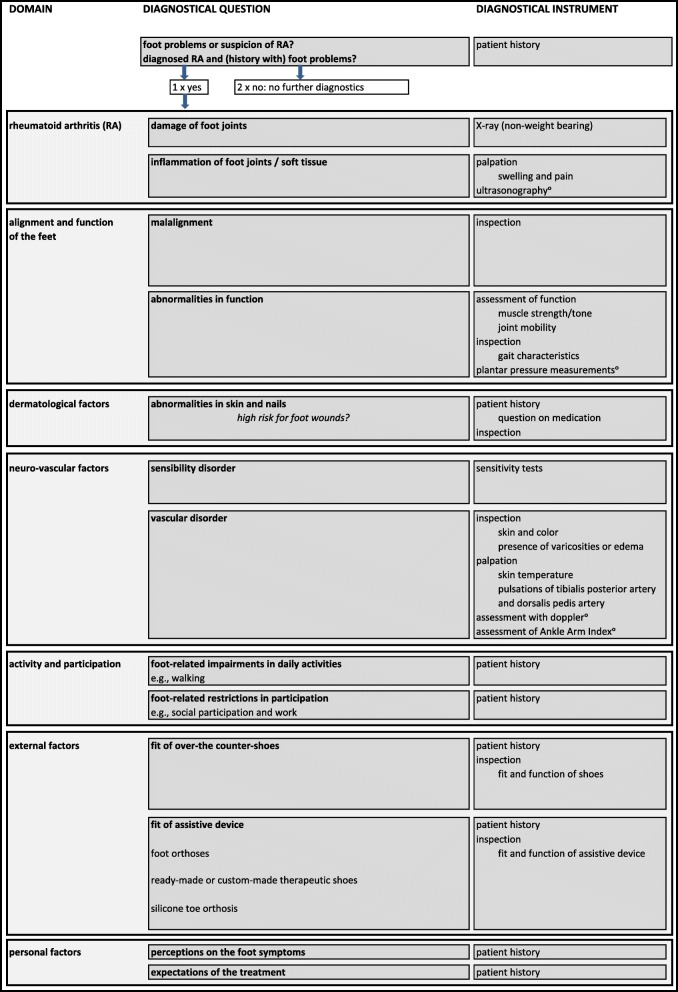
Framework for diagnosis of RA-related foot disease

The framework for diagnosis, as shown in Fig. 1, provides an overview of the different objectives in diagnosis of foot problems in RA and the corresponding instruments. Different objectives in diagnosis can be distinguished: i) detection of RA-related foot conditions; ii) medical diagnosis of RA; iii) (work-) diagnosis of foot function, dermatological factors, neuro-vascular factors, limitations in daily activities and restrictions in participation, external factors, and personal factors; and iv) monitoring of the progression of foot conditions/problems. For the Dutch situation, the role of the healthcare professions involved was described per objectives in diagnosis, as shown in Additional file 2.

**Fig. 2:**
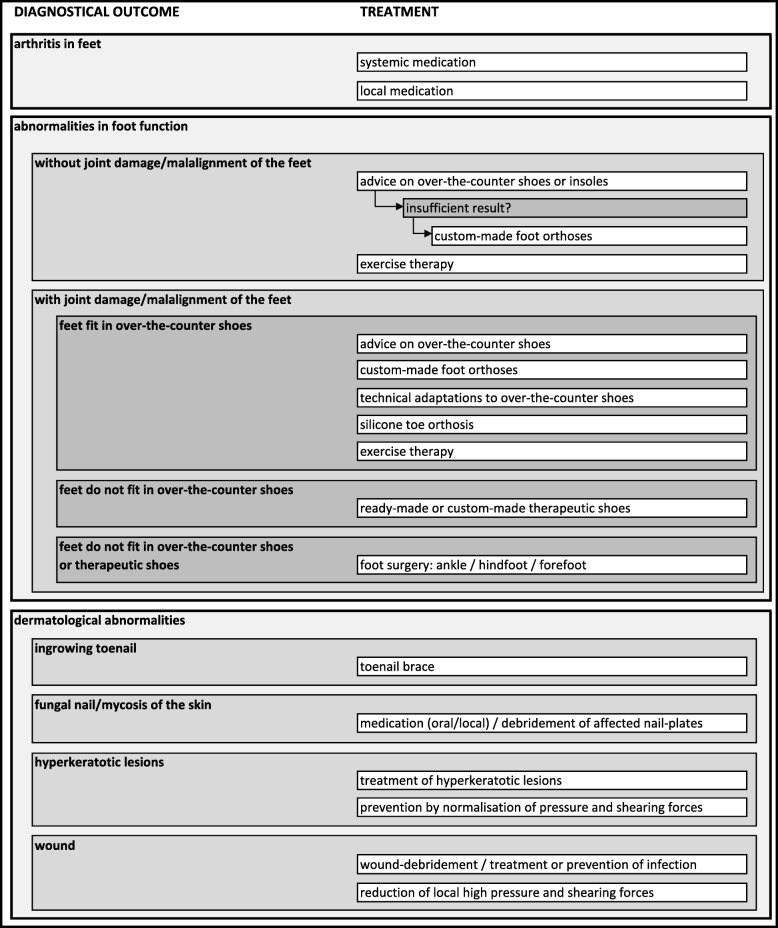
Framework for treatment of RA-related foot problems

The framework for treatment, as shown in Fig. 2, provides an overview of the treatment options for RA-related foot problems. The primary objectives in treatment are i) treatment of RA, ii) treatment of abnormal foot function, and iii) treatment of dermatological problems. In addition, treatment of neuro-vascular abnormalities should be considered. For the Dutch situation, the role of the involved healthcare professions was described per objectives in treatment, as shown in Additional file 3.

### Diagnosis

#### Check-ups of feet and shoes

Regular check-ups (for example annually) of the feet of people with RA are of great importance in detecting disease activity in an early stage. Especially because the most frequently used instrument to detect disease activity (with a 28 joint count [21]) excludes examination of the feet. Regular check-ups are also important in people with RA in remission, since pain and swelling of MTP joints are present in a substantial part of this patient group [22–24]. Long-term synovitis of foot joints can lead to joint damage and deformity [22]. Furthermore, check-ups of over-the-counter shoes worn by the patient are indicated. Malalignment of the feet is very common in people with RA and can cause pain during weight-bearing activities and difficulties with shoe-fitting. Inadequate shoe fit can lead to high local pressure and subsequent pain. The required fit and function of the shoes varies per person with RA. (Table 5) provides an overview of the developed recommendations on check-ups of feet and shoes.

#### Diagnostic imaging

Diagnostic imaging can be performed in addition to assessment of patient history and physical examination. Assessment of X-rays is an essential part of diagnosis of foot involvement (erosions and deformities of forefoot joints) by the rheumatologist. Ultrasonography can optionally be applied to detect and monitor foot involvement (synovitis in foot joints and inflammation of soft tissues). (Table 6) provides an overview of the developed recommendations on diagnostic imaging.

### Treatment

#### Medical treatment

Medical treatment primarily consists of the prescription of systemic medication by the rheumatologist. In addition, local medication can be applied in foot joints and soft tissues by corticosteroid injections. Furthermore, foot surgery can be performed to reduce pain and improve/maintain independent mobility, especially when a conservative treatment (neither medication nor surgery) is not successful or indicated. (Table 7) provides an overview of the developed recommendations on medical treatment.

#### Conservative treatment

Conservative treatment can be prescribed in addition to medical treatment. Conservative treatment can include therapeutic shoes, custom-made foot orthoses, exercise therapy, custom-made silicone toe orthoses, toenail braces, and treatment of toenails and skin. (Table 8) provides an overview of the developed recommendations on conservative treatment.

Therapeutic shoes can be prescribed in patients with abnormal foot function, damage/deformity of foot joints, or malalignment of the feet. Therapeutic shoes can be ready-made or custom-made. Ready-made shoes are i) over-the-counter shoes with technical adaptation, or ii) serially-produced shoes with extra depth, support, incorporated inlays, and optional technical adaptations [25, 26]. Custom-made shoes are developed for the individual patient based on specific measures and specifications, whereby a variety of technical adaptations can be incorporated [25, 26].

Custom-made foot orthoses can be prescribed to facilitate physical functioning by reducing pain and improving foot function [27–31]. In order to reduce pain and to improve foot function, the specific objectives of the foot orthoses can include i) normalising vertical plantar foot pressure, ii) reducing shear-forces acting on the feet, iii) correcting malalignment in feet with adequate joint mobility, and iv) supporting feet when correction is not indicated [27, 29, 32, 33].

Exercise therapy, in general, can be applied in people with RA to improve social participation and functioning in daily life [34]. Exercise therapy specific to the foot and ankle can be prescribed for the treatment of pain, muscle weakness, imbalance, and limited joint mobility [33]*.*

Custom-made silicone toe orthoses can be applied to i) correct a non-rigid abnormal toe-position and ii) to reduce local high pressure at the toes [35].

Toenail braces (made of surgical steel wire, titanium wire, or plastics, and attached to the nail with gel, acrylic, or composite) can be applied to improve the shape of the toenail by lifting the medial or lateral side [36]*.*

Treatment of toenails and skin can include treatment of i) nail fungus, ii) hyperkeratotic lesions, and iii) (pre-)ulcers or infections. Treatment of nail fungus consists of i) debridement of all hypertrophic and dystrophic nail-plates, ii) medication (oral or local), iii) patient-advice regarding the cause and treatment of the toenail fungus [32, 36]. In people with RA, prominent metatarsal heads are subject to high pressure and excessive shear forces during gait. These stresses stimulate the skin (stratum corneum) to produce hyperkeratotic lesions [32]. This can cause pain, corns, and wounds/ulcers [32, 36]. Scalpel or mechanical trimming techniques can be used to treat excessive hyperkeratotic lesions [36].

### Communication and organisation of RA-related footcare

Adequate communication between the patient and healthcare professional about the cause of foot problems, available treatment options, and anticipated outcomes are of great importance during the course of treatment. Understanding and involvement of the patient in determining the treatment strategy are important for adherence to the treatment and coping with the disease. Furthermore, specific advice on shoes and preventive and curative RA-related footcare is important for adequate self-management.

Healthcare professionals from various professions can be involved in the diagnosis and treatment of RA-related foot disease. The involvement of various professions depends on the severity of the foot problems, the work-field and expertise of the attending healthcare professionals, the organisation of footcare in the geographical area, and the preferences of the patient. Good communication and shared decision-making between the involved professionals is of great importance for adequate, multidisciplinary footcare in people with RA. (Table 9) provides an overview of the developed recommendations on communication and organisation of RA-related footcare.

## Discussion

These are the first published multidisciplinary recommendations specific to the management of foot problems in RA. The recommendations are based on the best available evidence and the opinions of experts with varying specialities and of patients. Forty-one recommendations (eight on diagnosis, 32 on treatment (of which four on communication) and one on organisation of footcare) were developed and approved by the expert group. In a recently published critical appraisal on clinical practice guidelines for the foot and ankle in RA, domains for foot and ankle management were identified [15]. The domains included in the previously published guidelines were multidisciplinary team care, access to foot healthcare, foot health assessment/review, orthoses/insoles/splints, therapeutic footwear, and other footcare treatments (patient education; corticosteroid injections; and treatment of hyperkeratotic lesions, wounds, and fungal infections) [15]. The present study covers these domains with up-to-date recommendations, based on literature and expert opinion. In addition, recommendations were developed on communication, foot surgery, exercise therapy, and the application of toenail-braces and provisional therapy (e.g. felt padding or taping) with clearly described contra-indications. The present recommendations address the total range of diagnostics and treatment options as applied in The Netherlands. Treatment of excessive callosities is recommended, although it is in contrast to the limited available evidence. One RCT showed no benefit of callus debridement over a sham procedure in terms of pain reduction, while sharp debridement may introduce potential risks [37]. Another RCT showed no-long term effect of sharp scalpel debridement on painful forefoot plantar callosities [38]. Despite this evidence, the expert group had the opinion that hyperkeratotic lesions can be treated, provided that the pre-defined contra-indications are taken into account. Moreover, for the Dutch situation, the role of the healthcare professions involved was described per objective in diagnosis and treatment. It should be noted that the present recommendations are aimed at managing RA-related foot problems in the Netherlands. Since the content, (expertise of) involved disciplines, and organisation of RA-related footcare may vary per country, this may hamper the generalizability of the frameworks and recommendations to other countries.

The level of evidence of the developed recommendations varies from 1 (highest) to 4 (lowest). Overall, most of the developed recommendations were based on expert opinion, as there is a lack of research evidence. Only a few number of the topics addressed in the recommendations were subject of investigation in previously published high-quality research. Evidence, based on randomised controlled trials’ (“RCT”) between-group differences, was found for the application of corticosteroid injections (in finger joints, based on a single RCT), foot orthoses (for treatment of pain and high forefoot pressure, based on multiple RCTs), ready-made therapeutic shoes (for treatment of high plantar pressure, based on a single RCT), patient education (not foot specific), and exercise therapy (not foot specific). A lower level of evidence (based on uncontrolled studies) was found for the application of ultrasonography, foot surgery, therapeutic shoes, silicone toe-orthoses, and toenail braces. Our findings clearly indicate that there are gaps in scientific literature on the management of foot problems in people with RA. More research is needed to strengthen the evidence on diagnosis and treatment of RA-related foot problems. Multiple areas with a lack of evidence were identified. The following topics for future research on diagnosis are indicated: i) diagnostic research on the psychometric properties, timing and frequency of ultrasonography for the detection of erosions and inflammation in the feet, and ii) the value of (yearly) check-up of the feet for the prevention or delay in progression of RA-related foot problems. For treatment the following topics for future research are identified: i) a definitive, high-quality RCT to investigate the effectiveness of corticosteroid injections in the foot, ii) RCTs on the effectiveness of different types of (fore-)foot surgery, therapeutic shoes, treatment of nails and hyperkeratotic lesions, and the comparative effectiveness of foot orthoses, and iii) development and evaluation of a foot-specific patient education program.

A multidisciplinary approach in the diagnosis and treatment of RA-related foot problems is recommended, as supported by several previously published guidelines [11, 12, 39–41]. Based on the opinion of the expert group, a multidisciplinary approach should consist of i) regular check-ups of the feet (for example annually) by a rheumatologist or a specialized nurse and, if indicated, ii) referral to another discipline (rehabilitation physician, orthopaedic surgeon, podiatrist, orthopaedic shoe-technician, pedicurist, or physical therapist). Referral should be considered when foot problems exist after reaching clinical remission [22–24, 42], when patients with increased disease states have mechanical foot impairments [5, 8], or when patients do not respond to or are ineligible for biological therapy and therefore continue to have active foot involvement [9]. Furthermore, adequate communication between the healthcare professionals involved and the patient (including shared decision-making and patient education) should be part of the treatment [43]. For example, in the prescription of therapeutic footwear communication and shared decision are of importance, especially to promote compliance of wearing them [44]. Adequate communication could be supported by a combined consultation with the professionals involved. In addition, (web-based) educational material may be helpful in patient education and could be developed within a network of specialised healthcare professionals or by patient organisations [45]. The healthcare professionals involved in, the access to, and the timing and content of management of foot problems may vary per country/geographical region. Therefore, developing and maintaining a network of specialised healthcare professionals, as well as developing a footcare pathway for diagnosis and treatment within this network are important steps in supporting multidisciplinary management [11, 12].

These are the first published multidisciplinary recommendations specific to the diagnosis and treatment of foot problems in people with RA. Expert opinions of several involved healthcare professions and patients (experienced in living with RA-related foot problems) were included in the recommendations. These national recommendations may be a first step towards developing international multidisciplinary recommendations for the management of foot problems in RA. The developed recommendations aim to contribute to i) uniformity in diagnosis, treatment, and guidance of people with RA-related foot problems; and ii) improved communication between, on the one hand, patient and treating healthcare professionals, and, on the other hand, between the healthcare professionals themselves. In future recommendations, the inclusion of more healthcare professions, such as general practitioners and physical therapists, who also have a role in RA foot management, could be considered. The development of the recommendations gave insight into the limited research evidence available on management of foot problems in RA. The gaps in literature could be topics for future research. Overall, more attention to RA-related foot problems in research is justified, as these are highly prevalent and have a substantial impact on patient quality of life.

## Conclusions

These are the first published multidisciplinary recommendations specific to the management of foot problems in people with RA. Multidisciplinary recommendations can provide guidance in timely referrals and access to adequate footcare. More research is needed to strengthen the evidence on diagnosis and treatment of RA-related foot problems. These national recommendations may be a first step towards developing international multidisciplinary recommendations for the management of foot problems in RA.

## Additional files

Additional file 1: Overview of the developed research questions and the used answering methods. (DOCX 43 kb)

Additional file 2: Framework for diagnosis with an overview of the role of the involved healthcare professions in the Netherlands. (DOCX 816 kb)

Additional file 3: Framework for treatment with an overview of the role of the involved healthcare professions in the Netherlands. (DOCX 774 kb)

## Abbreviations

ICF: International Classification of Functioning, Disability and Health
NRS-agreement: Numeric rating scale for agreement
RA: Rheumatoid arthritis
