# Supplementary material for: Multidisciplinary recommendations for diagnosis and treatment of foot problems in people with rheumatoid arthritis
Source: J Foot Ankle Res. 2018 Jul 4;11:37. doi: 10.1186/s13047-018-0276-z (PMC6030746; doi:10.1186/s13047-018-0276-z)
Supplement: Supplementary file 1 — Overview of the developed research questions and the used answering methods. (DOCX 43 kb) [file 13047_2018_276_MOESM1_ESM.docx]

**Additional file 1. Overview of the developed research questions and the used answering methods.**

| **Research-question** | **Method** |
| --- | --- |
| 1. Does the “Framework for diagnosis of RA-related foot disease” (Figure 1) provide an adequate overview of the different functions of diagnosis, detection, and monitoring of foot disease in patients with RA and the corresponding instruments? | - expert opinion |
| 1. Is an X-ray in a weight-bearing position or an X-ray in a non-weight-bearing position the preferred method for the detection of joint damage and joint deformity/malalignment in the feet? | - literature search (PubMed accessed April 13^th^ 2015)   ("radiograph*"[tiab] OR “x-ray”[tiab]) AND ("foot"[MeSH Terms] OR "feet"[tiab]) AND (“systematic review”[tiab] OR “review"[Publication Type])   - expert opinion |
| 1. Is ultrasound detection of foot arthritis the preferred method, above palpation? | - literature search (PubMed accessed April 29^th^ 2015)   ("ultrasonography"[tiab] OR "Ultrasound"[tiab] OR "ultrasonography"[MeSH Terms] OR "ultrasonics"[MeSH Terms] OR "ultrasonics"[tiab] OR "Sono graph*"[tiab]) and (inflam*[tiab] OR arthri*[tiab]) and ("foot"[MeSH Terms] or "feet"[tiab]) and ("system atic review"[tiab] OR "review"[Publication Type])   - expert opinion |
| 1. Which aspects should be included in individual shoe-advice on over-the-counter shoes for RA patients with foot disease? | - literature search (PubMed accessed May 4^th^ 2015)   (footwear [tiab] OR shoe*[tiab]) AND rheum* AND (“systematic review”[tiab] OR “review"[Publication Type])   - expert opinion |
| 1. Communication between patient and treating healthcare professional: which aspects on preventive and curative care should be included in patient advice? | - literature search (PubMed accessed April 13^th^ 2015)   (advise [tiab] OR communication [tiab] OR education) AND rheum* AND ("foot"[MeSH Terms] OR "feet"[tiab]) AND (“systematic review”[tiab] OR “review"[Publication Type])   - expert opinion |
| 1. Does the “Framework for treatment of RA-related foot disease” (Figure 2) provide an adequate overview of the potential treatment per diagnostic outcome? | - expert opinion |
| 1. What is the evidence on the effectiveness of a corticosteroid injection in the treatment of pain and impairment during walking? | - literature search (PubMed accessed May 5^th^ 2015)   (“corticosteroid” [tiab] OR “steroid” [tiab] OR “glucocorticoids”[tiab]) AND (” injection”[tiab] OR “intra-articular” [tiab] OR “intra-articular”[tiab] OR “local” [tiab]) AND ("foot"[MeSH Terms] OR "feet"[tiab]) AND (“systematic review”[tiab] OR “review"[Publication Type])   - expert opinion |
| 1. Is the application of a corticosteroid injection conducted by ultrasonography the preferred method, above the application of a corticosteroid injection without ultrasonography? | - literature search (PubMed accessed May 5^th^ 2015)   (“corticosteroid” [tiab] OR “steroid” [tiab] OR “glucocorticoids”[tiab]) AND (” injection”[tiab] OR “intra-articular” [tiab] OR “intra-articular”[tiab] OR “local” [tiab]) AND ("foot"[MeSH Terms] OR "feet"[tiab]) AND (“systematic review”[tiab] OR “review"[Publication Type]   - expert opinion |
| 1. What is the evidence on the effectiveness of foot surgery in the treatment of pain, impairment, and high local pressures? | - literature search (PubMed accessed May 5^th^ 2015)   (“surgery”[tiab] OR “operat*”[tiab]) AND ("foot"[MeSH Terms] OR "feet"[tiab] OR “ankle”[tiab] OR “ankles”[tiab] OR “metatars*”[tiab] OR “mtp*”[tiab] OR “forefoot”[tiab] OR “tars*”[tiab] OR phalang*[tiab] OR “toe”[tiab] OR “toes”[tiab] OR “hallux”[tiab] OR “midfoot”[tiab] OR calcane*[tiab] OR “heel”[tiab] OR “hindfoot”[tiab] OR “talus”[tiab] OR subtal*[tiab] OR talonavicul*[tiab] OR “tibia*”[tiab] OR “navicula*”[tiab] OR fibul*[tiab])AND (rheum*[tiab] OR arthri*[tiab]) AND (“systematic review”[tiab] OR “review"[Publication Type])   - expert opinion |
| 1. What is the evidence on the effectiveness of therapeutic shoes on foot function, foot pain, physical functioning, health-related quality of life, adherence, adverse events, and patient satisfaction in RA patients? | - literature search (PubMed accessed March 12^th^ 2015)   (("Arthritis, Rheumatoid"[Mesh] OR rheumatoid arthritis [tiab])) AND ("Shoes"[Mesh] OR shoe* [tiab] OR footwear* [tiab])   - expert opinion |
| 1. What is the evidence on the effectiveness of foot orthoses in the treatment of pain and impairment during walking? | - literature search (PubMed accessed May 6^th^ 2015)   ("Foot Orthoses" [MeSH Terms] **OR** "Orthotic Devices"[MeSH Terms] **OR** "Foot Orthoses" [tiab] **OR**  "inlay*" [tiab] **OR**  "Orthose*" [tiab]**OR** "Insole*"[tiab]) AND ("foot"[MeSH Terms] OR "feet"[tiab] OR “ankle”[tiab] OR “ankles”[tiab] OR “metatars*”[tiab] OR “mtp*”[tiab] OR “forefoot”[tiab] OR “tars*”[tiab] OR phalang*[tiab] OR “toe”[tiab] OR “toes”[tiab] OR “hallux”[tiab] OR “midfoot”[tiab] OR calcane*[tiab] OR “heel”[tiab] OR “hindfoot”[tiab] OR “talus”[tiab] OR subtal*[tiab] OR talonavicul*[tiab] OR “tibia*”[tiab] OR “navicula*”[tiab] OR fibul*[tiab])AND (rheum*[tiab] OR arthri*[tiab]) AND (“systematic review”[tiab] OR “review"[Publication Type])   - expert opinion |
| 1. What is the evidence on the effectiveness of exercise therapy in the treatment of pain and impairment during walking? | - literature search (PubMed accessed May 6^th^ 2015)   ("exercise*" [tiab] OR "stretch*"[tiab] OR “ therap*" [tiab]) AND ("foot"[MeSH Terms] OR "feet"[tiab] OR “ankle”[tiab] OR “ankles”[tiab] OR “metatars*”[tiab] OR “mtp*”[tiab] OR “forefoot”[tiab] OR “tars*”[tiab] OR phalang*[tiab] OR “toe”[tiab] OR “toes”[tiab] OR “hallux”[tiab] OR “midfoot”[tiab] OR calcane*[tiab] OR “heel”[tiab] OR “hindfoot”[tiab] OR “talus”[tiab] OR subtal*[tiab] OR talonavicul*[tiab] OR “tibia*”[tiab] OR “navicula*”[tiab] OR fibul*[tiab])AND (rheum*[tiab] OR arthri*[tiab]) AND (“systematic review”[tiab] OR “review"[Publication Type])   - expert opinion |
| 1. What is the evidence on the effectiveness of silicone toe orthoses and toenail braces in the treatment of pain and impairment during walking? | - literature search (PubMed accessed May 7^th^ 2015)   ("toe”[tiab] OR “toes”[tiab]) AND (“splint*" [tiab] OR "orthosis*"[tiab]) AND ("silicone*"[tiab])  (onychocryptosis[tiab] OR unguis incarnatus[tiab] OR ingrowing toenail*[tiab]) AND (orthonyxia [tiab] OR brace[tiab] OR treatment[tiab]) AND (“systematic review”[tiab] OR “review"[Publication Type])   - expert opinion |
| 1. What is the evidence on the effectiveness of the treatment of toenail fungus, calluses, gorges, and corns, wounds/ulcers, and of the application of pressure-reducing provisional therapies on pain and impairment during walking? | - literature search (PubMed accessed May 7^th^ 2015)   (callus[tiab] OR callosities OR hyperkeratosis[tiab] OR mycoses[tiab] OR onychomycoses[tiab]) AND (debridement [tiab] OR treatment[tiab]) AND ("foot"[MeSH Terms] OR "feet"[tiab] OR “toe”[tiab] OR “toes”[tiab] OR “plantar”[tiab]) AND (rheum*[tiab] OR arthri*[tiab]) AND (“systematic review”[tiab] OR “review"[Publication Type])   - expert opinion |
| 1. How can RA-related footcare be organised? | - literature: guidelines as detected in the systematic of Hennessy et al. [16] - expert opinion |
