# Supplementary material for: Multidisciplinary recommendations for diagnosis and treatment of foot problems in people with rheumatoid arthritis
Source: J Foot Ankle Res. 2018 Jul 4;11:37. doi: 10.1186/s13047-018-0276-z (PMC6030746; doi:10.1186/s13047-018-0276-z)
Supplement: Supplementary file 2 — Framework for diagnosis with an overview of the role of the involved healthcare professions in the Netherlands. (DOCX 816 kb) [file 13047_2018_276_MOESM2_ESM.docx]

**Additional file 2. Framework for diagnosis with an overview of the role of the involved healthcare professions in the Netherlands.**

***part 1***

**
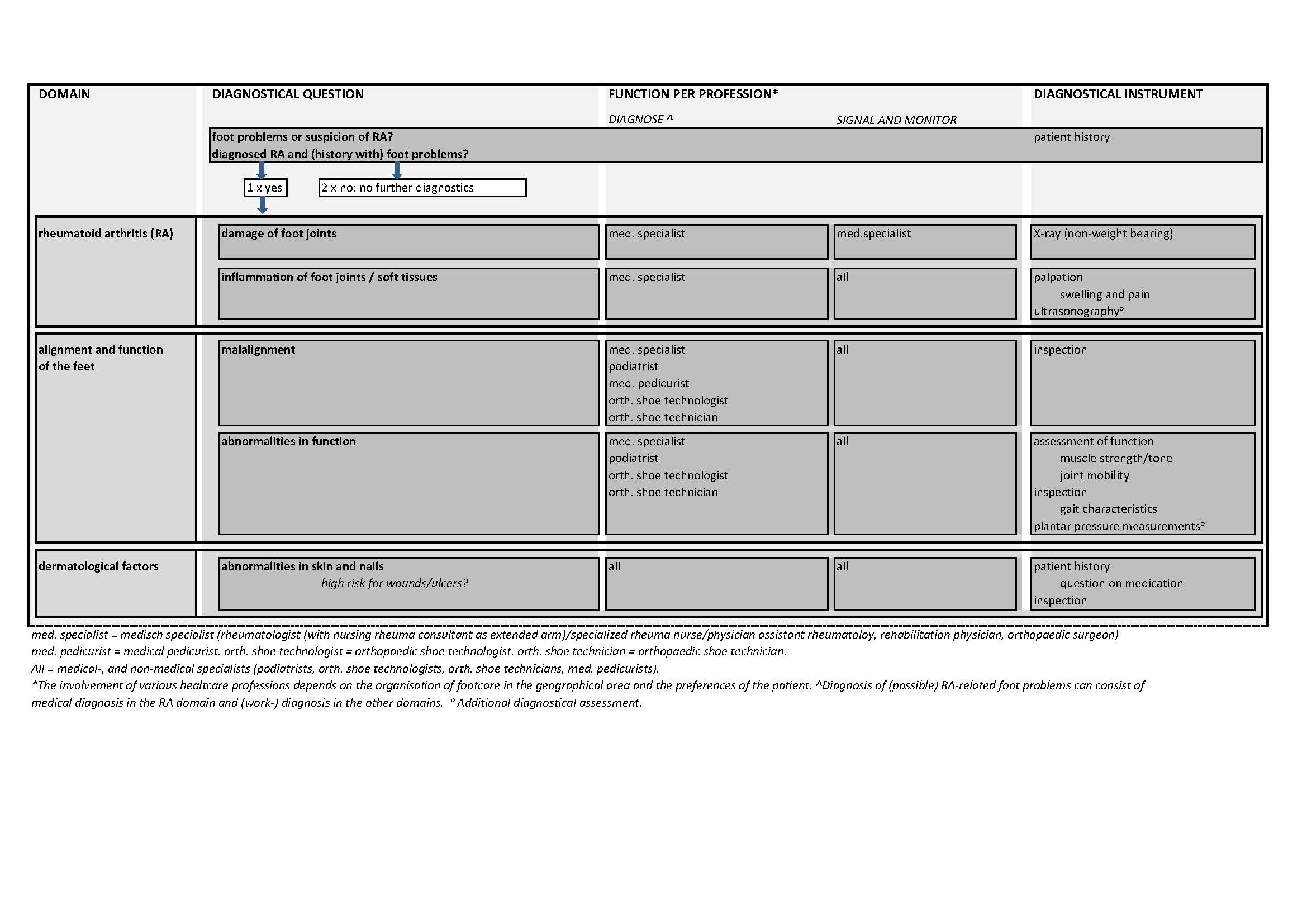
**

***part 2***

**
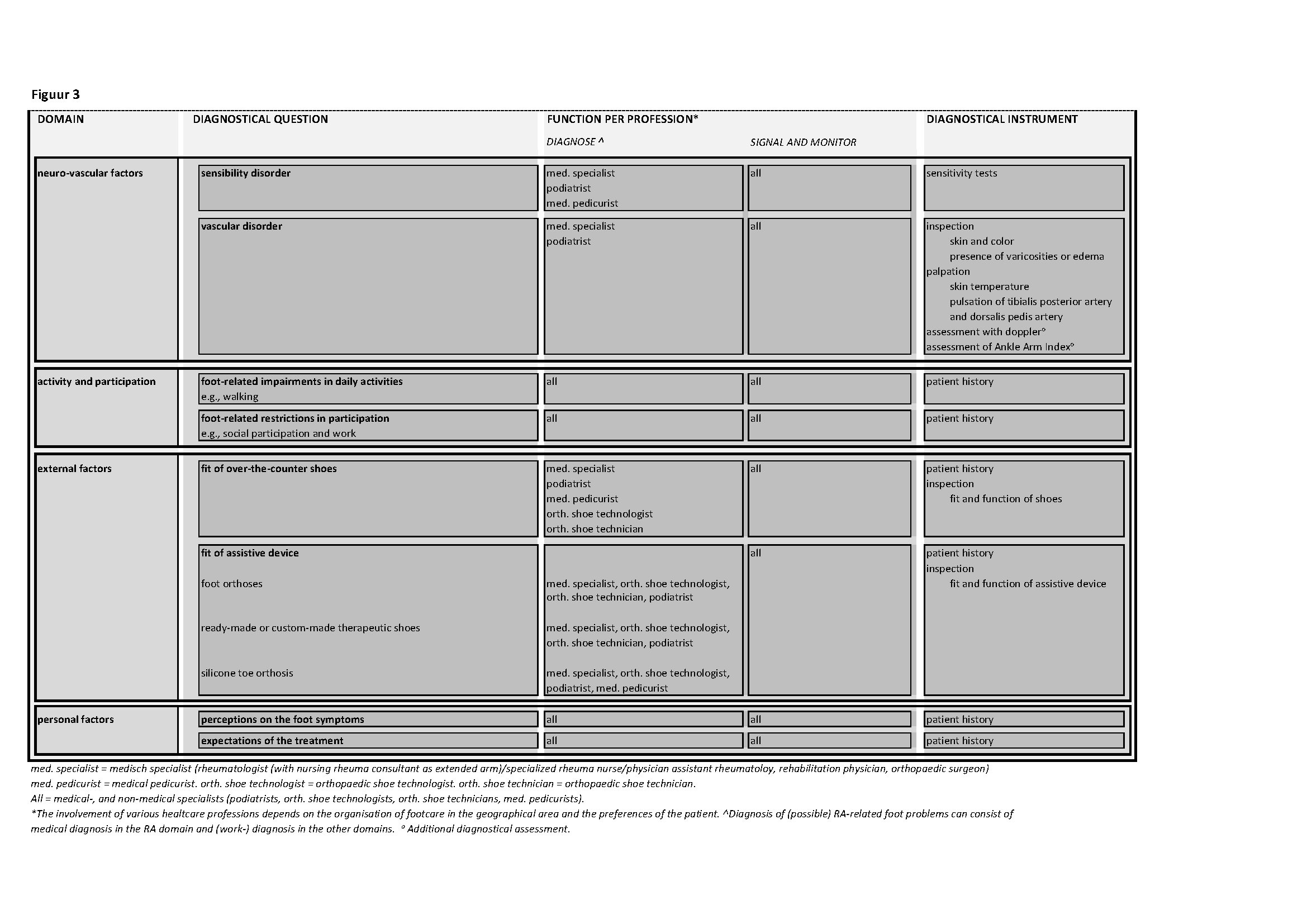
**
