# Supplementary material for: Multidisciplinary recommendations for diagnosis and treatment of foot problems in people with rheumatoid arthritis
Source: J Foot Ankle Res. 2018 Jul 4;11:37. doi: 10.1186/s13047-018-0276-z (PMC6030746; doi:10.1186/s13047-018-0276-z)
Supplement: Supplementary file 3 — Framework for treatment with an overview of the role of the involved healthcare professions in the Netherlands. (DOCX 774 kb) [file 13047_2018_276_MOESM3_ESM.docx]

**Additional file 3. Framework for treatment with an overview of the role of the involved healthcare professions in the Netherlands.**

***part 1***

**
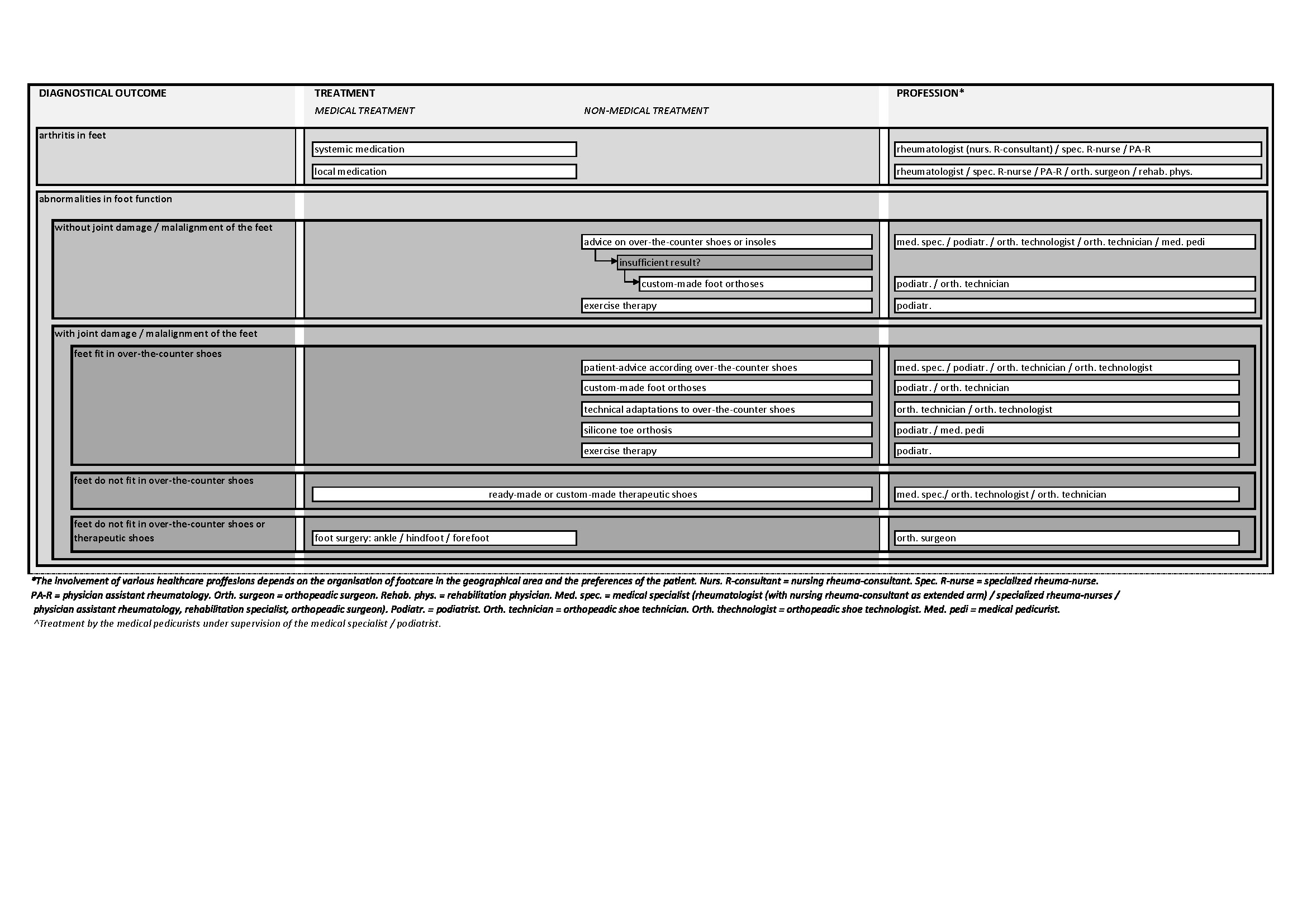
**

***part 2***

**
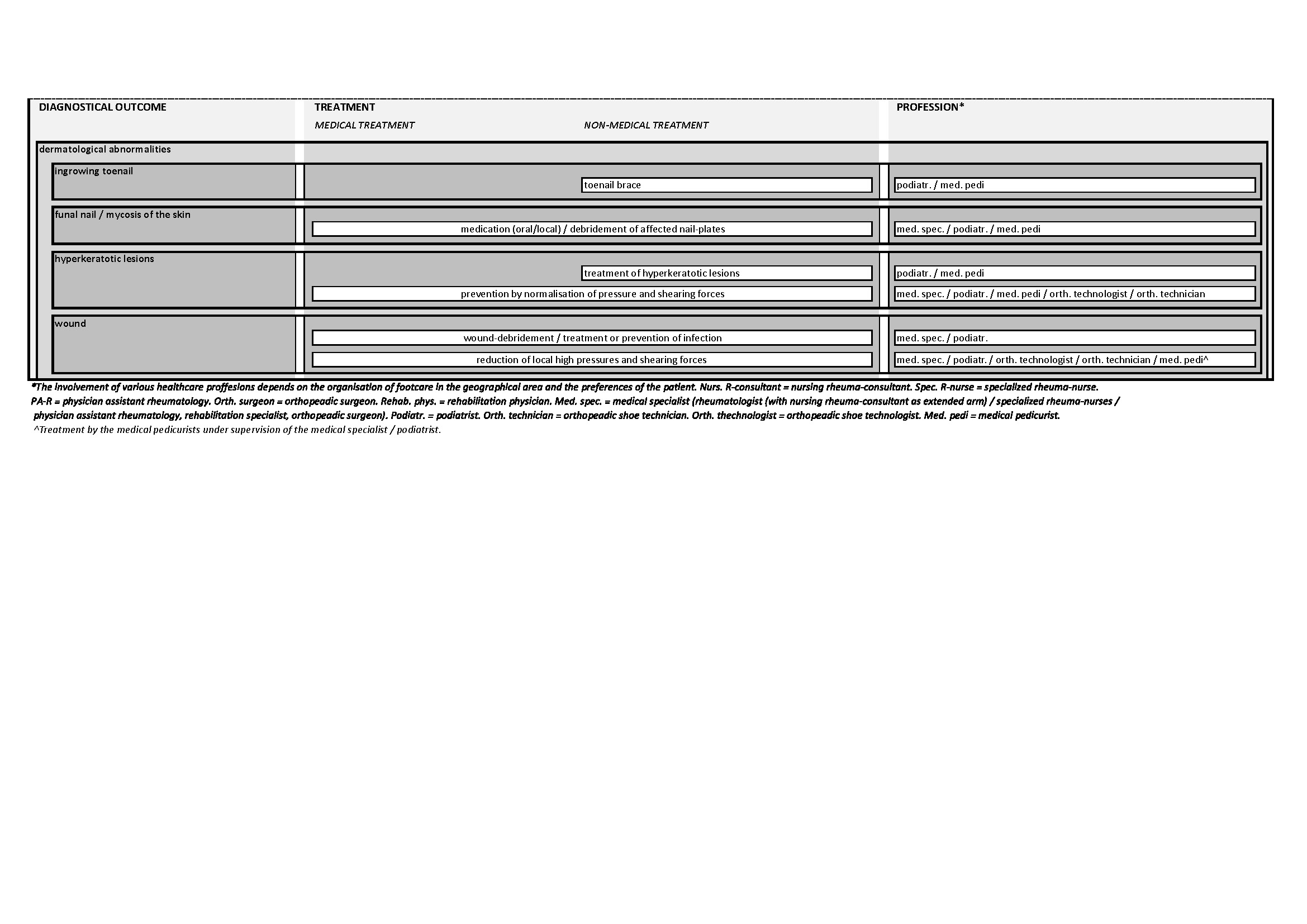
**
